# Comparing the Pathway to Success in European Countries Competing in the Swimming World Championships

**DOI:** 10.3389/fpsyg.2019.01437

**Published:** 2019-06-26

**Authors:** Inmaculada Yustres, Jesús Santos del Cerro, Fernando González-Mohíno, Michael Peyrebrune, José María González-Ravé

**Affiliations:** ^1^Department of Physical Activity and Sport Sciences, University of Castilla-La Mancha, Toledo, Spain; ^2^Department of Statistics, University of Castilla-La Mancha, Toledo, Spain; ^3^Departamento de Educación, Facultad de Educación y Lenguas, Universidad Nebrija, Madrid, Spain; ^4^Department of Exercise and Health Sciences, School of Sport, Exercise and Health Sciences, Loughborough University, Leicestershire, United Kingdom

**Keywords:** talent, swimming, progression model, youth, junior

## Abstract

**Purpose:** This study aimed to compare the performance progression model of the European countries that participated in the World Championships (WCs) from 2006 to 2017. Data from all championships were compared between the top five (1–5) and the next five (6–10) countries on the medal table. The study also identifies the ages of peak performance in senior swimmers, the annual ratio of progression and the effect of junior performance on senior success by these groups of countries. We hypothesized that: (1) countries with swimmers achieving better performances at the Junior WCs would also be higher in the medal ranking at the Senior WCs and (2) a higher annual performance progression in their swimmers increases the chances of countries being ranked in the top 5 at the Senior World Championships.

**Methods:** Participant’s data from all Junior and Senior WCs between 2006 and 2017 were obtained from FINA. The final, filtered database included 629 swimmers after removing those participating only in the Junior WCs and selecting swimmers from the top 10 countries. One-way ANOVA, F test, and decision tree methods were used to examine differences between the top (1–5) and the next best (6–10) countries on the medal table for first participation age, annual progress, and best-time in junior and senior championships.

**Results:** There was no difference (*p* = 0.492 and *p* = 0.97) between 1–5 and 6–10 ranked countries for best senior time and annual progression, respectively. Countries ranked in the top 5 at the Senior WCs had swimmers with faster times at the Junior WCs (*p* > 0.001). Decision tree analysis found that best-time at the Junior WC had the greatest explanatory capacity (94%).

**Conclusion:** European countries with swimmers who perform best at the Junior WCs are also likely to be in the top 5 countries that win medals at the Senior WCs.

## Introduction

The short (25 m pool) and long course (50 m pool) swimming World Championships (Cs) are run by FINA. Since 2001, the long course Championships have been held every 2 years in the odd years, while the long course after the Summer Olympics every 4 years. Since 2006, European (as a whole), American, and Australian swimmers are regularly the top nations ranked by number of medals won. Although the USA and Australia have been studied in a number of investigations (Trewin et al., 2004; Allen et al., 2014), little is known about the performances of the European countries. As nationality seems to play a key role in achieving the top positions at the WCs, a more detailed analysis of the performance of European countries is warranted.

Due to the increasing competition between nations for medals at major international events such as the World Championships and Olympic Games (Bosscher et al., 2006), many national sporting organizations have invested their available resources more effectively by identifying talented athletes well in advance (Vaeyens et al., 2009; Allen et al., 2015). Talent identification programs aim to identify athletes with high potential for success in senior elite sport (Allen et al., 2014). As a result, the best young athletes are routinely selected into talent development programs (Boccia et al., 2017) based primarily on their age-related competition performance (Lloyd et al., 2015). The concept of developing talent in youth is the goal of many coaches and sports systems. Developing talent at youth level to improve senior performance is the goal of many coaches and sporting organizations. Consequently, an increasing number of national governing bodies have adopted long-term development models in an attempt to provide a structured approach to the training of youth athletes (Svendsen et al., 2018).

Well-designed training plans can enhance performance by improving physiological parameters and enhancing technique (Morais et al., 2014). Details of these plans and their effects on performance are scarce. Many different factors contribute to performance in swimming (Morais et al., 2014) and other sports (Morais et al., 2017), although very little is known about their relative contribution, their progression as athletes develop and the interaction of these factors.

Longitudinal performance assessment is important to help coaches to define realistic goals and monitor training methods (Pyne et al., 2004). One way to achieve this is by tracking the swimmers’ performance for a given period of time and analyzing the progression between competitions and seasons. This information can be used to: (1) describe and estimate the progression and the variability of performance during and between seasons; (2) estimate chronological points that predict swimmer’s performances throughout their career or a given time frame; and (3) determine a swimmer’s probability to reach finals or win medals in important competitions (Costa et al., 2010).

If early sporting success is a pre-requisite for senior elite success (Neeru et al., 2013; Green, 2015), it is clear that maximizing sporting talent is an important goal of long-term development models. Some studies have described athlete development as an ascending scale and depicted improvements using a pyramid or linear model (Barreiros et al., 2014; Green, 2015). However, this assumption appears contentious given the low conversion rates of junior to senior athletes when focusing on specific groups (Durand-Bush and Salmela, 2002; Barreiros et al., 2014).

Consequently, the lack of studies that have focused on the general paths to success followed by international elite swimmers suggests there is a need to track the improvement and development of junior elite athletes. Performance models that provide useful information and minimize the drop out from junior to senior swimming would be extremely valuable (Costa et al., 2011; Allen et al., 2014). In addition, such predictive models can ensure that elite youth athletes are provided with a strategic plan to develop their maximal potential, thereby maximizing participation rates between junior and senior ages and improve long-term sports performance.

No studies have investigated trends in participation, age, and performance in European swimmers at the swimming WCs. In addition, the differences between the more successful and less successful teams have not been studied. The aim of the present study therefore was to compare the general performance progression model of the European countries that participate in the World Championships (WCs) from 2006 to 2017. Data compare the five best (1–5) and the next five best (6–10) European countries from a general medal ranking created with data from all the years analyzed. In addition, we identify the ages of peak performance in senior swimmers, the annual ratio of progression, and the effect of junior performance on senior success in these two groups of European countries.

## Materials and Methods

### Subjects and Design

Authors have no conflicts of interest to disclose. The Castilla-La Mancha University Ethical Committee approved this research dated November 30th 2016. This retrospective study was conducted with public data, and hence no informed consent was obtained. Results and birth dates were obtained from http://www.fina.org/ and http://www.omegatiming.com/ and processed by the authors. All historical data were retrieved from official results websites for the 2007, 2009, 2011, 2013, 2015, and 2017 Senior WCs and 2006, 2008, 2011, 2013, 2015, and 2017 Junior WCs. The age of the swimmers participating in Junior WCs must be between 14–17 and 15–18 years for women and men, respectively.

The final, filtered database (628 swimmers) included swimmers who swam in the Senior WCs (C3) and both Junior and Senior WCs (C1). The analysis was accomplished from top to bottom, trying to analyze where the swimmers who participate in the Absolute World Championship come from.

Mean ± standard deviations were identified by swim strokes, distances, and gender for a more appropriate standardization of the times. Each entry contains the full name, race time, position, age, country, gender, distance, swimming stroke, and year of competition. The distances analyzed were 50, 100, 200, 400, 800, and 1,500 m freestyle; 50, 100, and 200 m backstroke/breaststroke/butterfly, and 200 and 400 m individual medleys.

### Procedure

The times have been standardized by means of Ztime scores in order to compare swimmers’ times without influencing the variables gender, swim stroke, and distance. The, Ztime score was created by using the annual best performance of each swimmer.

$$Z_{ij}\;\;=\;\;\frac{X_{ij}-{\bar{X}}_{i}}{\sigma_{i}}$$

where *j* = individual *i* = group by gender, swim stroke, and distance.

The following variables have been defined within the model: the top five countries (1) European countries that have had the best results in medal rankings at the WCs (1–5: France, Italy, United Kingdom, Russia, and Sweden); and the next five (0) in the medal rankings (6–10: Hungary, Netherlands, Germany, Denmark, and Poland).

The criteria followed to subdivide the countries between the five best and 6th to 10th has been to develop a general ranking medal with the whole amount of European countries participating in the World Championships. For this purpose, we searched on internet the information about the ranking medal for each World Championship, and a general ranking was created integrating all this information. A total of 10 European countries had participated in all the World Championships analyzed in this study. Therefore, noticing the differences between the positions obtained by the five best and 6th to 10th countries, authors decided to subdivide the countries in that way.

The following variables were analyzed for statistical significance: (1) best-time Senior or peak performance at Senior level (BS): best-standardized performance in its senior stage; (2) minimum age (MA): age in years in which swimmers made their first World Championship; (3) best-time Junior (BJ): the best-standardized performance at the Junior competition; and (4) progress (P): an annual average of the interannual variations of standardized performances. As swimmers can participate in many events in a championship, the *z* scores of each of their performances were calculated and their minimum scores identified at Junior and Senior level. Both scores were then subtracted and divided by the number of championships.

### Statistical Analysis

Mean ± standard deviation was used to characterize swimmers from both groups (1–5 vs. 6–10 countries). Graphical and analytical descriptive statistical measures were used to identify differences between groups of countries in BS, BJ, and P. One-way ANOVA and F test were used to determine the differences and relationships between performances at the Junior and Senior WCs.

Non-parametric tests were also used to estimate the previous patterns with a classification methodology based on decision trees which enabled the identification of the significant aspects in order to achieve the best performances at the swimming WCs. The total sample was divided into a learning sample that was used to estimate both models and a test sample that allowed the estimated models to be validated. All analyses were performed with the software R.

## Results

Descriptive average values between the top five (1–5) and the next five (6–10) countries are shown in Table 1.

**Table 1:** Descriptive average values between top and non-top countries.

*N* = 628		
	Top countries	Non-top countries
% Swimmers	40.13	59.87
% Males	46.28	53.72
% Females	45.24	54.76
Mean age of best performance in senior	22.70 ± 3.50	22.15 ± 3.70
MA	21.11 ± 3.72	20.83 ± 3.81

“MA” minimum age: age in years in which swimmers involved in this study competed their first world championship competition.

**Figure 1:**
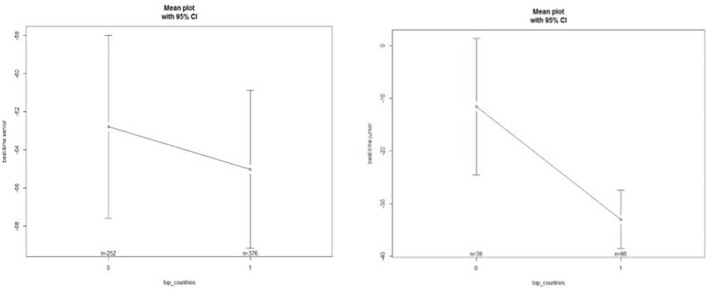
Differences between top and non-top countries in best-time senior and best-time junior.

No significant differences were found (*p* = 0.492) between the 1–5 and 6–10 countries (Figure 1).

As best time at senior level (BS) was not a discriminant factor in achieving 1–5 country status, further analyses were carried out to answer the hypothesis of the study.

We accept hypothesis (1) countries with swimmers achieving better performances at the Junior WCs would also be higher in the medal ranking at the Senior WCs as BJ was significantly higher in 1–5 vs. 6–10 countries (*p* < 0.001; *F* = 12.86, Figure 1).

**Figure 2:**
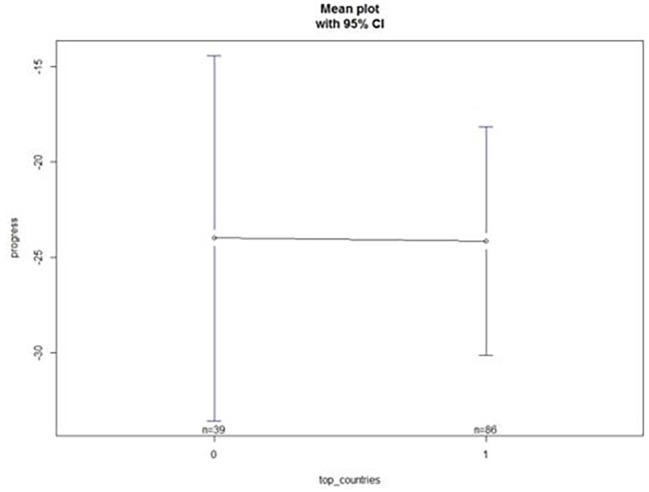
Differences between top and non-top countries in annual progression performance.

Despite this, we reject hypothesis (2) a higher annual performance progression in their swimmers increases the chances of countries being ranked in the top 5 at the Senior World Championships as no differences (*p* = 0.97; *F* = 0.001) were found (Figure 2).

The deviation analysis carried out by the decision tree model found that BJ had the greatest explanatory capacity (94%). In addition, both P and MA (3%) carry much less importance.

**Figure 3:**
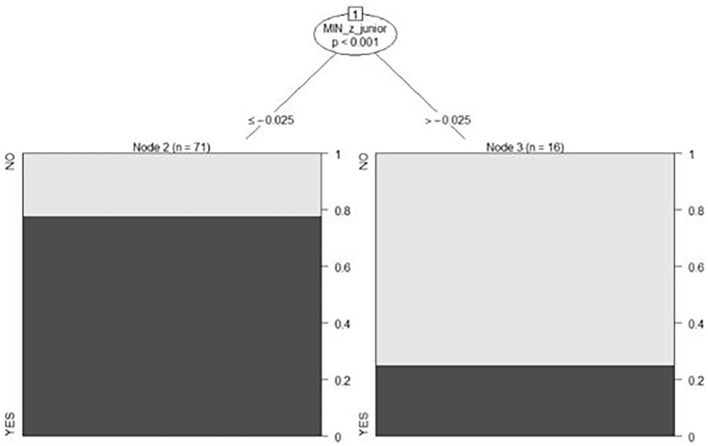
Conditional inference tree.

Therefore, the first relevant classification variable according to the estimated tree is BJ. If the score is below −0.025, there is a high probability (63%) of becoming one of the top (1–5) countries (Figure 3).

The confusion matrix, validation sample, and accuracy of the conditional inference tree are shown in Table 2.

**Table 2:** Confusion matrix with the validation sample.

Predicted		
Actual	No	Yes
No	1	10
Yes	4	23
Accuracy	0.63	

## Discussion

The main objective of the study was to compare the performance progression model of the top 10 European countries that participate in all World Championships (WCs) from 2006 to 2017. The swimmers’ performance in Junior WCs was significantly better in European countries ranked 1–5 than those ranked between 6 and 10 in the Senior WCs.

Previous studies have also suggested that Senior competitive level prediction increases markedly from the age of 16 (Costa et al., 2011; Neeru et al., 2013; Green, 2015). Svendsen et al. (2018) found that race performance at Junior level was found to be a strong predictor of subsequent success in senior elite cyclists. In swimming, Yustres et al. (2017) showed that 45.0% of Russian and 53.9% of Australian swimmers who achieved medals at the Junior WCs qualified for the finals in Senior WCs. Consequently, it seems that recruiting and developing swimmers from an early age will be advantageous in maximizing performance in Senior swimmers.

While this strategy appears logical, contradictory evidence from the German elite sport system showed that athletes recruited to talent-development squads at young ages exited the system the earliest, and the use of athlete support services was not substantially related to greater attainment of senior success. These observations were corroborated by Sokolovas et al. (2006), who found that most of the top American swimmers at the age of 17–18 were not ranked in the country’s top-100 at younger ages.

Allen et al. (2014) indicated that national swimming federations might improve the performance success by targeting resources towards larger groups of swimmers several years out from an international event. Concentrating on training processes and creating independence may help swimmers create an intrinsic motivation to succeed rather than an early specialized program that offers the benefit of early access to Sports Science and other resources.

Longitudinal performance prediction in National federations is increasingly popular to identify the characteristics necessary for winning medals at major events such as the World Championships or Olympic Games. Results from this study showed that an optimal annual progression in performance from Junior to Senior does not improve the chances of becoming a top-ranked country in the medal table at the Senior WCs. This indicates that on average, swimmers from all countries improve at a similar rate and the ones who start from a higher level at the Junior WCs are still in the top 5 at the Senior WCs. This supports the study by Trewin et al. (2004) who found no differences in mean progression rates between nations when analyzing the variability of competitive performance between FINA world-rankings and Olympic performances. This appears to be a conflicting area for research.

In contrast to our results, Pyne et al. (2001) found that lower ranked athletes with greater rates of performance improvement will increased their chances of winning a medal more than highly ranked athletes in future International competitions. Hopkins et al. (1999) also observed that an athlete in contention for a medal has to improve their performance by approximately one-half of the typical race to-race variation in performance (expressed as a standard deviation) to substantially increase their chances of success. In addition, improvements of this magnitude (~1% per year) should be considered when estimating performance times for future competitions (Pyne et al., 2004).

Longitudinal monitoring of performance progress must be able to differentiate between “normal” increases in performance caused by maturation and training, and “unnatural” improvement caused by doping (Hopker et al., 2018). Seasonal performance variability could become a useful indicator in targeting possible offenders. Previous studies suggested a coefficient of variation ranging from 1 to 1.5% in track and field athletics (Malcata and Hopkins, 2014) and 1% for elite rowing athletes (Smith and Hopkins, 2011). This confounding variable could be one reason why the annual rate of progression is not critical in becoming a top 5 country in the medal table at the Senior WCs.

In this study, there were no differences in minimum age (MA) between the top 5 countries and the next 5 on the WCs medal table. This suggests that national governing bodies would be advised to focus on maximizing swimmer’s performance at the Junior level over early exposure to Senior International competition.

Yustres et al. (2017) found a close relationship (*p <* 0.001) between the position obtained at Senior level and the number of years remaining competing in World Championships. Swimmers tend to be older when they achieve their best performance. It would appear that a greater number of experiences at international level will increase the chances of achieving better performances at the Senior WCs. It is likely that a better Junior performance level and competing for longer at Senior international level will increase the chances of success for counties aiming to be ranked in the top 5 at the Senior WCs.

In this study, we support the hypothesis that European countries with swimmers achieving an optimal performance at the Junior WCs have a better chance of success at the Senior WCs. We rejected the hypothesis that the swimmer’s annual performance progression is critical to Nations being a top-ranked European country in the medal table at the Senior WC. Future studies might analyze the evolution of the best junior swimmers competing the Junior World Championships to really determine how many will reach the World Championships of absolute category, identifying further contributory factors in helping countries to develop athletes and predict success at the Senior WCs. Besides, some other variables that could explain our main aim can be analyzed. However, it was not possible in our study due to the limited relevant information that we have about some other explicative variables.

## Conclusion

Countries with swimmers achieving an optimal performance in the Junior WCs will have a better chance of reaching the top 5 position in the medal ranking at the Senior WCs.

Average best time at the Senior WCs and an optimal annual progression performance from Junior to Senior do not affect the chances of becoming a top-ranked (1–5) country in the medal table at the Senior WCs.

This comparison between the performance progression model of European countries shows that countries aiming to reach the top positions in the medal should not focus on annual progression rates, the age of first performance, or the age of peak performance at Senior level. However, there is a 63% chance of swimmers who succeed at the Junior WCs that will also help their countries to achieve a top 5 medal ranking at the Senior WCs.

## Data Availability

Publicly available datasets were analyzed in this study. This data can be found here: http://www.omegatiming.com/index.htm.

## Author Contributions

IY, JG-R, and JS conceptualized, designed and performed the experiments. IY, JG-R, JS, and FG-M analyzed and interpreted the data. IY, JG-R, MP, and FG-M edited and critically reviewed the manuscript. IY, JG-R, MP, and JS wrote the manuscript.

### Conflict of Interest Statement

The authors declare that the research was conducted in the absence of any commercial or financial relationships that could be construed as a potential conflict of interest.
